# Time for gender mainstreaming in editorial policies

**DOI:** 10.1186/1758-2652-14-11

**Published:** 2011-03-08

**Authors:** Shirin Heidari, Mirjam J Eckert, Susan Kippax, Quarraisha Abdool Karim, Papa Salif Sow, Mark A Wainberg

**Affiliations:** 1 Journal of the International AIDS Society, 1216 Cointrin, Geneva, Switzerland; 2 Social Policy Research Centre, University of New South Wales, Sydney, Australia; 3 Centre for the AIDS Programme of Research in South Africa, Nelson R Mandela School of Medicine, University of KwaZulu-Natal, Durban, South Africa; 4 Department of Epidemiology, Columbia University, New York, New York, USA; 5 Department of Infectious Diseases, University of Dakar, BP 5005, Senegal; 6 McGill University AIDS Centre, Jewish General Hospital, Montreal, Quebec, Canada

## Abstract

The HIV epidemic has been continuously growing among women, and in some parts of the world, HIV-infected women outnumber men. Women's greater vulnerability to HIV, both biologically and socially, influences their health risk and health outcome. This disparity between sexes has been established for other diseases, for example, autoimmune diseases, malignancies and cardiovascular diseases. Differences in drug effects and treatment outcomes have also been demonstrated.

Despite proven sex and gender differences, women continue to be underrepresented in clinical trials, and the absence of gender analyses in published literature is striking. There is a growing advocacy for consideration of women in research, in particular in the HIV field, and gender mainstreaming of policies is increasingly called for. However, these efforts have not translated into improved reporting of sex-disaggregated data and provision of gender analysis in published literature; science editors, as well as publishers, lag behind in this effort.

Instructions for authors issued by journals contain many guidelines for good standards of reporting, and a policy on sex-disaggregated data and gender analysis should not be amiss here. It is time for editors and publishers to demonstrate leadership in changing the paradigm in the world of scientific publication. We encourage authors, peer reviewers and fellow editors to lend their support by taking necessary measures to substantially improve reporting of gender analysis. Editors' associations could play an essential role in facilitating a transition to improved standard editorial policies.

## Editorial

The HIV epidemic is increasingly affecting women, and the proportion of women living with HIV globally has been continuously on the rise. According to UNAIDS' latest epidemic update, women constitute slightly more than half of all people living with HIV. In sub-Saharan Africa, women bear a disproportionate burden of HIV infection: nearly 60% of adults living with HIV are women, and young women, 15 to 24 years of age, are eight times more likely to be infected than men in the same age range. This trend is not restricted to sub-Saharan Africa. Women living with HIV also outnumber men in the Caribbean. In Asia, the proportion of HIV infections in women has been increasing: from 21% in 1990 to 35% in 2009. Similarly, the proportion of women with HIV in countries where injecting drug use has been the main driver of the epidemic has substantially increased [1].

Biological and social differences between men and women influence health risk and disease progression. Women appear to be biologically more susceptible to heterosexual HIV transmission partly due to the large mucosal surface of the vagina and cervix as potential viral entry points [2]. Other factors also suggested to play a role are micro-abrasion during sex, levels of immunosuppression in the vaginal tract achieved by seminal fluid, and morphological changes during the hormonal cycle or hormonal fluctuations. In addition, women can also be exposed through anal sex [3].

For a given CD4 cell count and disease stage [4], it has been shown that women have a comparatively lower level of virus, indicating that women may progress at a lower viral load than men [5]. Furthermore, women record higher CD4 cell counts at varying stages, including seroconversion, AIDS diagnosis and death from AIDS-related illnesses [6].

Despite evidence that antiretroviral drugs are equally effective in women and men, different toxicity profiles have repeatedly been reported. For example, in a recent study in Uganda, being female was shown to be associated with stavudine-related toxicities [7]. Studies of pharmacokinetics and pharmacodynamics of antiretroviral therapies (ARTs) have noted important variations between women and men, demonstrating higher plasma concentration in women as compared with men for several drugs, including atazanavir, lopinavir, saquinavir, nevirapine and efavirenz. Even differences in drug clearance rate have been reported, for example, nevirapine and saquinavir are shown to have a lower clearance rate in women [8-10].

So far, these known differences have not translated into different clinical management, with the exception of nevirapine, which is not recommended for women with CD4 cell counts above 250 cells/mm^3 ^due to a higher risk of hepatotoxicity [11]. However, clinical implications of such differences remain to be further explored, and additional investigations are required to fully map the sex-based differences in HIV disease progression, treatment outcome and clinical management.

It is important to note that in addition to physiological and biological factors, social and behavioural factors play a fundamental role in a person's health and in the observed health disparities between men and women. Women have an increased vulnerability to HIV because of gender-based power relations: they suffer domestic and other gender-based violence, and in gender-subordinate positions, condom use is difficult, if not impossible, to negotiate [12,13]. Together with socio-economic disadvantages, such as illiteracy and poverty, these factors seriously impede women's access to testing, counselling, prevention and treatment programmes.

Gender analysis is essential in demonstrating how each sex's biological processes interact with gender's social roles, socio-economic context, health-seeking behaviours and access to healthcare systems. Such analyses are especially important in low-income countries where gender differences in certain populations can have dramatic effects on life choices and opportunities, and in subpopulations, such as transgender people, who are particularly vulnerable.

However, despite proven sex and gender differences, women continue to be underrepresented in clinical trials, and gender analyses of data continue to be conspicuous by their absence in published literature. Women have historically been excluded from clinical trials for a number of reasons. Concerns about causing harm to the foetus have often been cited as the primary reason. However, there are other contributing factors that have practical implications. Desire to minimize heterogeneity in a study population is one. Studies powered sufficiently to detect differences in subgroups would be larger, more costly and less feasible (though not impossible). Furthermore, hormonal variations during the menstrual cycles introduce additional variables that may complicate the analysis of the effect of a treatment [14].

Disregard of sex-specific data analysis and generalization of research findings from one sex to another result in interventions that are not firmly based on evidence for efficacy in women and could potentially lead to suboptimal treatment and poorer outcomes. Ensuring participation of women and other subpopulations, such as transgender, in research, including collection and appropriate analysis of data, is crucial in drawing accurate conclusions that can be applied to all subpopulations.

In 1993, following articulated concerns by women's health advocates about exclusion of women from clinical trials, the US National Institutes of Health (NIH) issued its revitalization act that mandates all NIH-funded clinical trials to include women and minorities in adequate numbers to be able to identify valid differences, if any, between sexes and racial subgroups (USA, Public Law 103-43). The authors know of no such law or mandate in other countries. Despite the effort by the NIH to ensure rightful participation of subpopulations in trials, women continue to be underrepresented in clinical trials. In 2002, a study indicated that only 24% to 25% of participants in many phase I and II clinical trials in the United States were women [15]. A more recent study, in 2006, showed that women remain underrepresented in randomized controlled trials supported by US federal funding. In 46 clinical studies that included both male and female participants, women represented, on average, 37% of the study population and only 24% when limiting the analysis to drug trials [16].

In recent years, there has been an emergence of initiatives that advocate for gender mainstreaming in health and life science research, in particular in the HIV field. The UNAIDS initiative, "Make HIV Trials Work for Women and Adolescent Girls", highlights the importance of establishing a "new norm" of research, where women and adolescent girls are included in drug intervention studies. In addition, data disaggregated by sex and also age are key to this initiative as different factors over a women's lifespan need to be considered [17]. Resources that compile important information and make access to knowledge more widely available are gaining momentum. One such resource can be found on the website, http://www.whatworksforwomen.org, which "provides strategies and evidence on a full range of gender-sensitive programming for women and girls".

In 2010, on the occasion of International Women's Day, the International AIDS Society, jointly with 15 other organizations, including UN agencies, civil society partners and pharmaceutical companies, released a Consensus Statement, "ASKING THE RIGHT QUESTIONS: Advancing an HIV Research Agenda for Women and Children". The Consensus Statement highlighted 20 research recommendations, calling for increased investments and efforts to fill these gaps. The statement called in particular for research data "to be disaggregated by sex to ensure opportunities for gender-based analysis using a variety of indicators, such as retention in ART programmes, morbidity and mortality, loss to follow-up, and pharmacokinetic and pharmacodynamic parameters" [18].

On the occasion of International Women's Day 2011, we would like to encourage these efforts to be extended to the world of scientific publishing. Despite increased efforts and advocacy to include women and girls in clinical trials, provision of sex-disaggregated data and gender analysis in most published literature is rare and often an exception to the rule. Geller *et al *showed that 87% of studies of randomized clinical trials published in nine influential medical journals in 2004 "did not report any outcome by sex or include sex as a covariate in modelling" [16]. There is little evidence that the trend has changed since then.

Increasing the involvement of women, and improving the provision of gender analysis remains an urgent health and women's rights priority. Collection, analysis and reporting of sex-disaggregated data are paramount in ensuring that women and men have equal opportunities to participate and enjoy the highest attainable standards of physical and mental health; this is one of the fundamental human rights, as recognised in the Universal Declaration of Human Rights and International Covenant on Economic, Social and Cultural Rights.

Investigators, ethical review boards, funding bodies, pharmaceutical industry, regulators, peer reviewers and journal editors should play an active and leading role in a broad range of aspects, from study design to guidelines and recommendations.

It is imperative that editors and publisher show bold leadership in changing the paradigm in the world of scientific publishing. Instructions for authors issued by journals contain many guidelines for good standards of reporting, and a policy on sex-disaggregated data and gender analysis should not be amiss here. It is encouraging to see high-profile journals take up this cause, such as *Nature *raising this issue in June 2010 [19], although concrete actions in this direction through formal requirements in editorial policies have yet to be realized.

The *Journal of the International AIDS Society *is proud to take a first step in this direction and feature such a policy on its website (http://www.jiasociety.org/info/about/) encouraging our authors to consider sex and gender differences in their study designs and requiring that gender analysis is presented in submitted manuscripts where applicable. Inclusion of this section in our journal's instructions for authors is currently under negotiation with the publisher. We welcome peer reviewers in lending their support by ensuring that the aspect of gender is included in their overall assessments of a manuscript and highlighting the absence of it when necessary.

We call on fellow editors and publishers to take appropriate measures to improve the reporting of sex-based analyses in peer-reviewed publications. Editors' associations play an essential role in continuously striving to improve standard editorial policies, and should follow suit. Joint effort is necessary to ensure that women, just as much as men, reap the fruits of research.

## Competing interests

SH is an employee of the International AIDS Society, and her salary is provided partly by unrestricted educational grants from the following pharmaceutical companies: Abbott, Boehringer Ingelheim, Gilead, Merck, Pfizer, Roche, Tibotec and ViiV Healthcare. MJE, SK, QAK, MAW and PSS have no competing interests to declare.

## Authors' contributions

SH wrote the first draft of the article. SK, QAK, MAW, PPS and MJE provided critical comments and edited the article. All authors read and approved the final manuscript
